# Influence of Strontium on the Biological Behavior of Bioactive Glasses for Bone Regeneration

**DOI:** 10.3390/ma16247654

**Published:** 2023-12-15

**Authors:** Amanda Vieira Silva, Déborah dos Santos Gomes, Rayssa de Sousa Victor, Lisiane Navarro de Lima Santana, Gelmires Araújo Neves, Romualdo Rodrigues Menezes

**Affiliations:** 1 Graduate Program in Materials Science and Engineering, Federal University of Campina Grande, Campina Grande 58429-900, PB, Brazil; amanda_vieirasilva@hotmail.com; 2 Laboratory of Materials Technology (LTM), Federal University of Campina Grande, Campina Grande 58429-900, PB, Brazil; rayssavictor1@gmail.com (R.d.S.V.); lisiane.navarro@professor.ufcg.edu.br (L.N.d.L.S.); gelmires.araujo@professor.ufcg.edu.br (G.A.N.)

**Keywords:** bioactive glass, strontium, bone regeneration, biological behavior

## Abstract

Bioactive glasses (BGs) can potentially be applied in biomedicine, mainly for bone repair and replacement, given their unique ability to connect to natural bone tissue and stimulate bone regeneration. Since their discovery, several glass compositions have been developed to improve the properties and clinical abilities of traditional bioactive glass. Different inorganic ions, such as strontium (Sr^2+^), have been incorporated in BG due to their ability to perform therapeutic functions. Sr^2+^ has been gaining prominence due to its ability to stimulate osteogenesis, providing an appropriate environment to improve bone regeneration, in addition to its antibacterial potential. However, as there are still points in the literature that are not well consolidated, such as the influence of ionic concentrations and the BG production technique, this review aims to collect information on the state of the art of the biological behavior of BGs containing Sr^2+^. It also aims to gather data on different types of BGs doped with different concentrations of Sr^2+^, and to highlight the manufacturing techniques used in order to analyze the influence of the incorporation of this ion for bone regeneration purposes.

## 1. Introduction

Bioactive glass (BG) has been commonly studied as a bone substitute due to its outstanding biological properties, such as great binding to newly formed bone tissue and the deposition of a carbonated hydroxyapatite layer on its surface that promotes bone regeneration [1]. The first BG reported in the literature was initially studied by Hench et al. [2], BG (45S5^®^), which allows the artificial organ to effectively fit into the bone cavity [3] and has been gaining space in clinical applications such as Perioglas^®^ [4] and Ceravital^®^ [5]. Its first application in orthopedics was approved by the FDA in 2000 [6].

These materials stimulate bone gene expression and angiogenesis, promoting excellent osteosynthesis, bioavailability, biodegradability and cellular conductivity [7]. However, these materials have some disadvantages, such as a small processing window, promoting to crystallization during the production process and negatively influencing bioactivity [8,9]. Furthermore, the original BG composition contains a large amount of sodium [10], and so could lead to cell death [11]. Therefore, there is a need for more research into the use of BGs in orthopedics and traumatology.

One of the attractive characteristics of bioactive glass is its ability to incorporate inorganic therapeutic ions (ITIs) (such as strontium, copper, zinc or fluorine) into its structure. These ions can promote an increase in the biological functions of bioactive glass, favoring bone formation due to their stimulating effects [12]. Knowledge about the effects of ions on the body leads to increased interest in their use, mainly in biomaterials. However, the quantity determined so that the effect is not harmful generates caution even though they are efficient in wound healing and bone regeneration [13].

In recent years, the positive effects of strontium (Sr^2+^) on bone metabolism have made it widely recognized as an appropriate material for use in bone tissue engineering. It was verified that the addition of the Sr^2+^ ion, even in small amounts, can promote the stabilization of the bone structure [14], stimulating osteoblast activity, reducing osteoclast activity [12,15] and enabling the formation of new bone tissue. Furthermore, Sr^2+^ is able to promote effective antibacterial action, having an inhibitory effect on several strains, such as *Escherichia coli* and *Porphyromonas gingivalis* [16,17]. Therefore, strontium ranelate and strontium chloride have been commonly used in the treatment of osteoporosis and bone pain [14,18].

The literature has reported the potential of Sr^2+^ in increasing alkaline phosphatase (ALP) activities, collagen synthesis and the expression of osteoblastic markers, leading to increased osteogenesis [19]. However, the long-term systemic use of Sr^2+^ is associated with serious adverse reactions, such as the risk of myocardial infarction, thromboembolic events and severe skin reactivity, with its indication being limited to the treatment of severe osteoporosis by the European Drug Administration in 2013 [20,21].

Additionally, pharmacokinetic studies have shown that when orally administered, the bioavailability of Sr^2+^ is less than 20%, making its release preferential at the site of the bone defect. This makes the doping of materials with Sr^2+^ a desired possibility for systemic administration in the treatment of osteoporotic bone fractures [22].

Other studies have also shown that small amounts of Sr^2+^ (2.5% mol) exhibit inhibitory effects against the bacteria *Staphylococcus aureus (S. aureus*), *Escherichia coli (E. coli*) and *Porphyromonas gingivalis (P. gingivalis).* However, this effect depends on the degradation rate of the samples, as well as the pH of the medium.

In this sense, studies addressing Sr-doped BG are of great relevance as BG allows the local release of Sr^2+^, avoiding negative reactions associated with its long-term systemic use [20]. However, there are still challenges related to Sr-containing BG, since an in-depth study of the incorporated amounts of this element is necessary, as high amounts of Sr can have negative effects on the biological properties of BG. These include inhibiting many osteoclastic cells and compromising bone tissue regeneration and remodeling. This effect can lead the tissue to a condition of osteonecrosis and can make the bones brittle [23].

Furthermore, despite the increasing number of studies on BG containing Sr^2+^, there are still gaps regarding certain issues related to its influence on the biological behavior of BG. In this sense, this review aims to describe relevant information about the state of the art of the biological behavior of bioactive glasses containing Sr^2+^, gathering data on different types of Sr-doped BG compositions, and analyzing their characteristics and applications since there are different doping types and behaviors that are considered positive or negative depending on their use.

## 2. Bioactive Glasses

Bioactive glasses are amorphous materials based on silica, boron, or phosphor, considered biocompatible, bioactive and osteoconductive. The first bioactive glass reported in the literature was produced in the 1970s by Larry Hench and collaborators, named Bioglass^®^ 45S5 (45S5 Bioglass, 46.1% SiO_2_, 24.4% NaO, 26.9% CaO and 2.6% P_2_O_5_, in mol%) [2]. This was the first material that did not promote the formation of fibrous tissue on its surface after implantation, inducing an interfacial connection with the host tissue [2,24,25]. Bioglass^®^ was released for clinical use in 1984 by the Food and Drug Administration, resulting in several useful applications. In the 1990s, particulate Bioglass^®^ became available for clinical applications as PerioGlas^®^ [4].

In general, the basic composition of these materials consists of oxides that are classified according to their function. Network-forming oxides are those composed of non-metallic elements that have the function of forming a glassy network through multiple bonds with oxygen (O), such as SiO_2_, B_2_O_3_ and P_2_O_5._ Network modifier oxides are those composed of alkaline elements and alkaline earth, such as Na_2_O and CaO. And the intermediate oxides are those used when there is a need to modify the physical or chemical behavior of the material, such as Al_2_O_3_ and ZnO [26].

**Figure 1:**
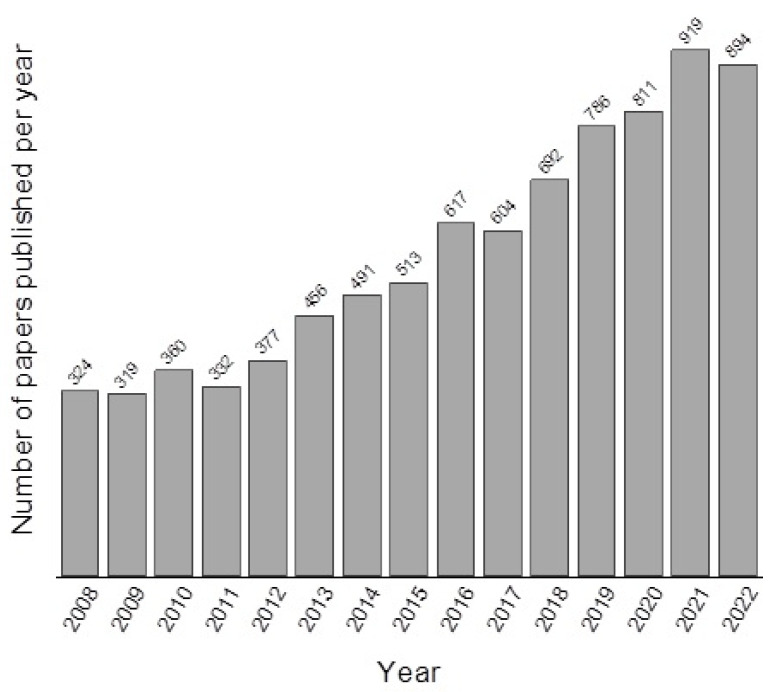
Number of studies published with bioactive glass in the last 14 years according to the web of science—search topic “bioactive glass”.

A common characteristic of bioactive glasses is the kinetic modification of their surface over the implantation time. When in contact with biological fluids, the formation of a biologically active layer of carbonated hydroxyapatite (HCA) is observed on its surface, which promotes better interfacial interaction with neighboring tissues [23,27,28,29]. Since the HCA phase formed is chemically and structurally similar to the mineral phase of bone, osteoblasts can proliferate on this apatite layer, differentiating and forming an extracellular matrix composed of biological apatite and collagen. Therefore, when the host bone tissue comes into contact with this layer, the connection between the biomaterial and the natural bone tissue will occur chemically [30]. Therefore, BG particles have been used in biomedical applications since 1985 [31] and have continued to be widely studied in recent years (Figure 1).

Many studies show that bioactive glasses promote enzymatic activity [32,33] and vascularization [34,35], maintain osteoblastic adhesion, regulate the growth and differentiation of mesenchymal cells into osteoblasts [36], and also exhibit outstanding in vivo biocompatibility properties [37].

Diverse methods has been reported in the literature for the synthesis of bioactive glass powders, fibers and scaffolds. Among them, we can mention melt quench [38,39], which consists of the fusion of oxides and subsequent tempering in a graphite mold or water; sol–gel [40,41,42], in which precursors are added to a solution for the hydrolysis step, then the sample is condensed and the colloidal sol is produced, with the gelation process occurring a few hours later; hydrothermal processes [43,44], which consist of a closed system containing the heterogeneous solution under high temperature and in the presence of aqueous solvent and pressure; electrospinning [45,46,47]; and solution blow spinning [48]. In the first process, a solution is subjected to an electrical charge, overcoming surface tension, and producing a jet of fluid that allows the evaporation of the solvent and results in fibers deposited in a collector. In the second, the solution is ejected through a system in which an air jet directs it to a collector where the fibers formed by the evaporation of the solvent will be deposited. Laser-spinning [49,50], flame spray [51,52], microemulsion [53], polymer foam replication [54,55] and 3D printing [56,57,58] are also examples of techniques used. Some of these processes are used in synergy and in all of them the BGs produced can have different diameters, surface area, degradation rate or bioactive potential, justifying the choice of each technique depending on the desired result.

These studies have shown that, according to the chemical composition, glasses with different characteristics and with different levels of reactivity with tissues could be produced. Thus, several glass and glass–ceramic compositions have been developed to improve the properties and clinical abilities of bioactive glass, with the possibility of incorporating different therapeutic ions in the structure of these glasses being reported, in order to improve their physical characteristics and generate favorable biological and therapeutic effects.

## 3. Bioactive Glasses Containing Strontium

Several ITIs have been incorporated in bioactive glasses, such as lithium (Li^+^), boron (B^3+^), magnesium (Mg^2+^), strontium (Sr^2+^), silver (Ag^+^) and zinc (Zn^2+^). Many of them have been shown to be able to induce osteoblast precursor differentiation through growth factor signaling pathways or to stimulate other processes to support new tissue growth [59]. Among them, Sr^2+^ has stood out in recent years and has been the focus of several studies in bone tissue engineering as it plays an important role in bone formation [60].

Strontium is an alkaline earth metal (2A) that was discovered in the 18th century as a result of lead mining in Scotland [61]. It is an abundant element in ocean water, groundwater and the earth’s crust, which can also be found in the human diet in leafy greens, grains and seafood [62,63,64]. Additionally, Sr^2+^ is a biologically beneficial trace element that is also abundant in human tissues, with its amount in the skeleton being approximately 0.335% of its calcium (Ca) content. This element has structural, physical and chemical similarity to Ca, and due to its affinity to bone tissue, Sr isotopes have been widely used and studied during the last half century in tissue engineering for the treatment of bone-related diseases.

In 1870, the physiological function of Sr^2+^ was analyzed for the first time. It was observed that this ion was naturally incorporated into the bones of animals that were fed this element [65], which generated interest in further research on its effects on other organs. Research has observed that the ion was naturally incorporated into the bones of animals that were fed this element. In 1952, the Sr^2+^ was used for therapeutic purposes for the first time. It was observed that patients with osteoporosis whose treatment consisted of a combination of Sr^2+^ lactate with a Ca supplement achieved skeletal remineralization (Shorr, 1952), promoting an increase in bone mass, a reduction in bone pain and an increase in mineralization. Also, since the 1940s, Sr-89 has been used to treat bone pain in patients with metastatic bone cancer, as an adjunct to chemotherapy, radiation and hormone therapy [66].

Despite the few studies carried out with human beings, the research available to us confirms the therapeutic potential of materials doped with Sr^2+^, indicating their high biocompatibility, degradability and improved osteogenic abilities [67,68,69,70]. Furthermore, the presence of Sr^2+^ can promote a reduction in the degradation rate of doped BGs compared to pure BGs. This reduction is useful as it allows adequate time to promote extracellular matrix formation during the bone tissue regeneration process [70,71].

Additionally, studies performed on animals have revealed that the use of strontium chloride in a low concentration induces an increase in bone formation [72,73,74]. These works have provided evidence for the use of Sr^2+^ as an anti-osteoporotic agent, sparking interest in the study and subsequent applications of this ion.

**Figure 2:**
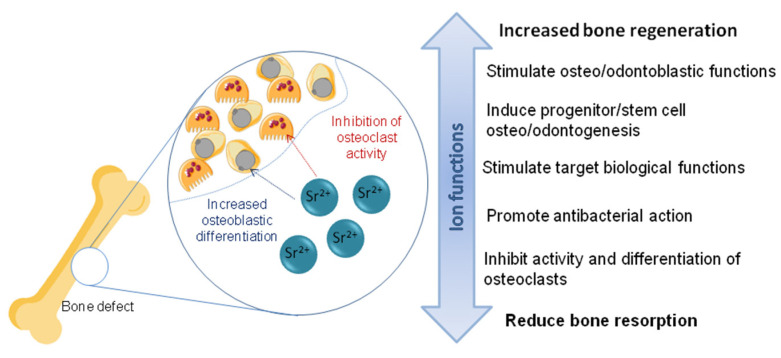
Effect of Sr^2+^ ion on bone tissue repair.

In general, research has shown that Sr^2+^ promotes significant effects on hard tissue repair processes (Figure 2), such as the stimulation of osteo/odontoblastic functions and induction of osteo/odontogenesis of progenitor/stem cells [75]. Sr^2+^ can reduce bone resorption by inhibiting osteoclast activity and differentiation, in addition to increasing pre-osteoblastic cell replication and osteoblastic differentiation, promoting the formation of new tissue [60,76]. As a result, it is widely used clinically, in the form of strontium ranelate (SrR) for the treatment of osteoporosis in recent decades [77,78], and in toothpastes for repairing teeth [79]. When internalized into cells, Sr^2+^ interacts with intracellular signaling molecules, being able to enhance and stimulate biological functions. This process is known and studied through bone morphogenetic protein 2 (BMP2) [78].

In general, bone healing is a process induced by the immune system in which immune cells can reach effector cells through paracrine mechanisms, being able to alter pathological alveolar bone resorption and physiological bone regeneration. For example, macrophages are crucial immune cells that play a critical role in the initial period of bone repair and host defense after biomaterial implantation [80]. When internalized into cells, Sr^2+^ interacts with intracellular signaling molecules, being able to guide the organism toward favorable osteogenesis, modulating macrophages in the bone tissue microenvironment [78,80].

In mesenchymal stem cells (MSCs) the process of osteogenic differentiation is more complex and involves different cells such as osteoprogenitors, pre-osteoblasts, osteoblasts and osteocytes, in addition to various types of intercellular and intracellular signaling transduction, such as signaling pathways, transcription factors and growth factors. Receptors such as peroxisome proliferator-activated γ2 (PPARγ2) and transcription factors such as Runt (Runx-2) are fundamental genes to promote lipogenic differentiation and osteogenic bone marrow mesenchymal stem cells (BMSCs) [81].

Research has shown the influence of Sr^2+^ on the reduction in adipocyte genes such as PPARγ2 and CEBPα, and the induction of osteogenic genes such as Runx2, ALP, osteocalcin (OCN) and bone sialoprotein (BSP). this results in the activation of regulatory kinases via extracellular signaling (ERK)-MAPK and Wnt, which directly influence the increase in bone deposition and the reduction in adipogenesis, improving bone tissue regeneration. Choi and Park [82], in their research, proved that the presence of Sr^2+^ on the surface of acid-blasted/etched titanium oral implants (SLA) is favorable for the initial functions of macrophage cells and increases the osteogenic capacity of the SLA surface. Sr^2+^ promoted an increased expression of the regenerative phenotype of macrophages and the production of anti-inflammatory cytokines IL10, while suppressing the inflammatory cytokine TNFα. It regulated chemical markers, while reducing the expression of adipogenesis-related transcription, in addition to promoting early cellular events in ST2 stem cells, such as cell spreading and critical integrin gene expression, increasing osteogenic differentiation.

In the same year, Zhang et al. [83] studied the influence of Sr^2+^ incorporation in BGs on the proliferation and osteogenic differentiation of mouse mesenchymal stem cells (mMSCs). And they showed that mRNA expressions and protein expressions involved in the NFATc and Wnt/β-catenin signaling pathways were all upregulated, indicating synergistically increased osteogenesis, while Wang et al. [84] produced calcium silicate ceramics incorporated with strontium (Sr-CS) and observed that Sr promoted improvement in the regeneration of osteochondral defects, improving the induction of osteogenesis and chondrogenesis of MSCs. In vivo transplantation showed that Sr-CS scaffolds distinctly improved cartilage and subchondral bone regeneration, when comparing pure calcium silicate samples. This effect was attributed to increased Sr^2+^ in the osteogenic and chondrogenic differentiation of MSCs.

Research has also demonstrated the double effect of Sr^2+^ on bone metabolism [85,86,87]. And studies have shown that this ion inhibits apoptosis, which occurs through the ATK pathway, in addition to encouraging adherence, and the differentiation of osteoblasts that promote the deposition of a new cellular matrix through the α2 and β1 integrin pathways [85,86]. It can also positively regulate the expression of osteogenic genes, such as ALP, OCN and BSP, through the activation of mitogen-activated protein kinase (MAPK) signaling and the phosphorylation of ERK1/2 [88]. In osteoclasts, through the expression of the SOST gene, Sr^2+^ stimulates the production of sclerostin, which has the function of inhibiting osteogenesis and increasing bone mass [85,86]. Furthermore, it can reduce the expression of genes related to osteoclastogenesis, such as tartrate-resistant acid phosphatase (TRAP) and matrix metalloproteinase-9 (MMP-9), reducing the resorption activity of bone tissue [87].

The Sr^2+^ is often inserted into the vitreous lattice as a modifier cation. Many studies show the development of BG based on silicate, phosphate and borosilicate, in which Ca is partially replaced by Sr^2+^ [89,90,91]. However, research indicates that the introduction of Sr^2+^ in percentages higher than CaO will promote a proportional increase in the silica/calcium content, generating greater connectivity and promoting structural changes in the BGs network [92].

The replacement of Ca by Sr in the BG network presents good biological capacity, since the radius and ionic charge are similar [93]. In their study, Dai et al. [94], using BG HX-BGC (12–45SiO_2_-10–35P_2_O_5_-5–48CaO_2_-5–15Na_2_O-3.5–4.9SrO, % by weight), evaluated the effect of the presence of Sr^2+^ on the composition of glass. And they observed that Sr replaced part of the Ca and increased the interplanar distance and lattice parameters due to the larger ionic radius of the Sr element (1.16 A) compared to Ca (0.94 A), which enlarges the glass lattice. The BGs produced showed good HA deposition capacity and greater apatite stability.

Sr-doped BGs are commonly manufactured using the sol–gel method or the melt quench method, as they provide advantages in the productivity and chemical flexibility of the BG [95]. Sr-doped BGs produced via sol–gel showed good osteogenic capacity in rat calvaria bone defects, as well as improved antibacterial activity against *Staphylococcus aureus* and *Escherichia coli* [96], while those produced by melt quench could increase the apatite formation and control the osteoblast and osteoclast activity when compared to non-doped BG [97]. Furthermore, it has also been shown that glasses doped with Sr^2+^ produced by melt quench have antibacterial activity against E. coli and a positive effect on the viability of Saos-2 cells (ATCC^®^ HTB-85™ human osteosarcoma cell line) [98]. Therefore, these behaviors confirm that Sr-doped BGs produced using sol–gel and melt quench have a promising impact on the study of bone regeneration.

According to Marie and Hott [74], the Sr^2+^ incorporation in a borate bioactive glass promoted a delay in the kinetics of boron release, inducing better adhesion of SaOS-2 cells (that are similar to osteoblasts) and improving glass cytocompatibility. In addition, the formation of multilayers of apatite is observed on its surface, which may induce the formation of new tissue.

Studies carried out in recent decades have brought positive evidence regarding the use of Sr^2+^ in bone remodeling. This justifies the increase in the incorporation of this ion in a variety of materials used in bone implants. It was also observed that the Sr-doped BGs demonstrated better performance than their Sr-free counterparts, in studies aimed at bone regeneration. In this sense, the influences of Sr^2+^ on the biological behavior of bioactive glass are summarized in Table 1.

Analyzing existing patents is also important because observing the research groups in which patents are developed allows us to understand which groups of researchers intend to develop the next generation of commercial materials. However, in the patent literature, there is still a significant gap in the bibliographic analysis of Sr-doped BGs. Through a search carried out on “Google patents” using the term “bioactive glasses” and refining with “strontium”, it was possible to find a few patents that involve only this material. These patents are presented in Table 2.

### 3.1. In Vitro Behavior

Over the last years, in vitro studies have been devoted to analyze the potential application of Sr-doped bioactive glass in bone repair due to its ability to bind to living bone and its good bioactivity, biodegradability and osteoconductivity. According to Araujo et al. [105], BG scaffolds of 45S5 (46.1SiO_2_–2.6P_2_O_5_–24.4Na_2_O–26.9CaO) doped with Sr^2+^ 2%.mol, produced by melt quench, showed cell viability greater than 90% using the NCTC clone 929 cell line. Also, Zhang et al. [112] produced mesoporous BG (57.2SiO_2_–7.5P_2_O_5_–35.3(SrO + CaO)) doped with 5, 10 and 15 mol% Sr^2+^, via a hydrothermal process, and observed increased proliferation, alkaline phosphatase (ALP) activity and gene expression related to the osteogenesis and mineralization of ECM from MC3T3-E1 cells. In another study, Solgi et al. [113] produced BG (SiO_2_–CaO–SrO–P_2_O_5_) using sol–gel and observed in in vitro experiments with osteosarcoma cells (MG-63) that the replacement of CaO by SrO with 5% of Sr^2+^ revealed optimal cell proliferation and stimulated alkaline phosphatase (ALP) activity.

Mosaddad et al. [71] evaluated the potential application of 45S5 BG scaffolds doped with collagen and Sr^2+^ in bone regeneration and observed that the Sr^2+^ presence promoted better stimulation and induction of cell growth. After 14 days of testing, this material showed higher ALP activity and higher Ca content. Cell viability assessed by MTT indicated the cell growth of human mesenchymal stem cells, hMSCs, on the surface of pure BG and BG-Sr (5 mg/mL), with survival rates of around 80%. Also, according to the results of DAPI (Diamidino-2-phenylindole), greater cell binding and proliferation were observed for samples doped with Sr^2+^, confirming the greater biocompatibility of the BG-Sr structure for cell differentiation and regulation.

Cell proliferation and osteogenic differentiation experiments performed by Zhang et al. [104] indicated that the ideal amount of Ca replacement for Sr^2+^ in BG 45S5 produced by a melt quench process was 10 mol%. This replacement content was able to stimulate the proliferation capacity of BMSCs with greater ALP activity and showed the highest expressions of the four main osteogenic markers (BMP-2, OPN, BSP and OCN), when compared to concentrations of 0, 5 and 20 mol% Sr^2+^. Furthermore, the results of Ca deposition activity and alizarin red S stain confirmed that 10% Sr^2+^ doping promoted the osteogenic differentiation of OVX-BMSCs.

The bioactivity and chemical reactivity of bioactive glass in bone regeneration applications are associated with the ability to bind to soft and hard biological tissues through the development of the apatite layer, stimulating cell adhesion, proliferation and differentiation. Many studies correlate this characteristic to the material’s ability to create bonds through ion exchange in simulated body fluid (SBF) after a series of chemical reactions, spontaneously forming a biological apatite layer on their surface [23,64].

The chemical composition of BGs, the production method and their morphological characteristics considerably influence the biomineralization process [14,114]. In this sense, it is worth mentioning that the addition of some network-forming oxides can significantly reduce in vitro apatite deposition with biomineralization [115], inhibiting the growth of apatite layers due to the possibility of hindering the dissolution of the glass and the release of the ions [116]. Ions such as Sr^2+^ can affect the crystallization kinetics, crystallinity and thermal stability of the system to devitrification [117] and thus influence the glass biomineralization.

Used as a network modifier, SrO partially replaces CaO due to its similar chemical role. This replacement can promote changes in the BG network depending on both the synthesis method and the composition [118]. A higher replacement of CaO by SrO increases the silica/CaO content in mole percentage, resulting in greater network connectivity [92]. This has been observed in the range of 1–20 mol% of Sr^2+^ substitution produced by melt quench [119,120]. Furthermore, the Sr addition can promote the formation of new crystalline phases that are less reactive and therefore less bioactive than the amorphous phase [118], generating BG devitrification and reducing the apatite formation rate [118,121].

Regarding the synthesis methods, glasses produced by melt quench made it possible to retain the amorphous structure after sintering [121], while the sol–gel-derived glasses showed that the increase in the Sr^2+^ content promoted an increase in the crystallization of the material [122].

Some studies have already shown that bioactive glasses derived from the melt quench process and Sr-doped (with concentrations in the range of 1–5 ppm) promote a faster deposition of the apatite layer on their surface, making them more bioactive when compared to non-doped glasses [68,112]. According to O’donnell and Hill [92], it also showed that this may be related to the increase in reactivity caused by the replacement of Ca by Sr, which generates a slight expansion of the silica network and increases the dissolution rate. However, the effect of introducing Sr^2+^ in BGs on the formation of the apatite layer remains a non-consolidated question in the literature.

In BGs produced via sol–gel, it was observed that the addition of up to 5% of Sr^2+^ promotes an improvement in the formation of the hydroxyapatite layer. In this sense, Swe et al. [103] produced semi-crystalline S53P4 bioglasses (SiO_2_–P_2_O_5_–Na_2_O–CaO–Al_2_O_3_–SrO) doped with 3 mol% Sr^2+^ via sol–gel. When Ca was replaced by Sr, the presence of the Sr ion promoted an expansion in the network, proven by the displacement of the BG diffraction peaks due to the larger size of the Sr^2+^ dopant ion when compared to the Ca host ions. A high density of HAp crystals was observed in a needle shape after immersing the Sr-doped BGs for 7 days, when compared to pure BG. In another study, Ranga et al. [16] studied the biomineralization ability after the introduction of Sr into the bioactive glass matrix of a silver-doped amorphous SiO_2_-CaO BG (55SiO_2_–40CaO–(5 − x)SrO–xAg_2_O) produced using sol–gel. Samples were immersed in SBF, with the formation of a hydroxyapatite layer on the surface of the doped BG being observed after an immersion time of 7 and 14 days. Recently, Bahati et al. [106] produced BGs nanoparticles doped with Sr^2+^ (74SiO_2_–(26 − x) CaO–xSrO, x = 0, 1, 3 and 5) using the sol–gel method to evaluate their ability to form the hydroxyapatite layer and dissolve the glass. As higher concentrations of Sr^2+^ were incorporated into the BG, the particle size decreased by up to 17% when compared to the pure BG sample, which could directly affect its properties. The formation of the hydroxyapatite layer was observed after 3 and 7 days of immersion of the samples in SBF, but the mineralization decreased with increasing Sr concentration.

This same trend was shown by researchers when producing Sr-doped amorphous BG produced by fusion and hydrothermal processes. For example, Fredholm et al. [68] reported that when replacing Sr with Ca (0–100 mol%) in amorphous BG (SiO_2_–P_2_O_5_–CaO–Na_2_O) produced by melt quench, an increase in apatite formation and an expanded glass network were observed. Also, Yin et al. [101] produced borate-based bioactive glass (18SiO_2_–36B_2_O_3_–22CaO–6Na_2_O–8K_2_O–8MgO–2P_2_O_5_) doped with Sr via the melt quench method. After immersing the BG with 6 mol% Sr^2+^ in SBF for 7 days, a nanostructured hydroxyapatite layer on the surface was formed, increasing the BG biomineralization ability. Furthermore, El Baakili et al. [43] studied the kinetics of the formation of the apatite layer on the surface of binary bioactive glasses (63SiO_2_–(37 − x)CaO–xSrO) doped with Sr^2+^ (x = 0.2–1 mol%) and prepared via a hydrothermal process. Their results highlighted the positive effect of Sr incorporation on BG biomineralization due to its physical and chemical similarity with calcium ions, showing a greater formation of the apatite layer on the surface of the material.

On the other hand, bioactive glasses produced by melt quench with contents greater than 10 mol% of SrO replacing CaO showed a significant decrease in the formation of the apatite layer. Goel et al. [123] produced amorphous BG doped with Sr^2+^ ((36.07 − x)CaO–xSrO–19.24MgO–5.61P_2_O_5_–38.49SiO_2_–0.59CaF_2_, x = 0 and 10 in mol%) via melt quench and reported a decrease in the apatite formation capacity of glasses containing Sr. They attributed the results to a greater strength of the metal–oxygen bond (351 kJ.mol^−1^ for Ca–O and 389 kJ.mol^−1^ for Sr–O) and lower electronegativity of Sr^2+^ (0.99) when compared to Ca^2+^ (1.04), which consequently reduces the ability of Sr^2+^ to be exchanged with H^+^ in the solution to promote the formation of the apatite layer. Massera and Hupa [120] produced semi-crystalline BG S53P4 (53.85SiO_2_–22.66Na_2_O–1.72P_2_O_5_–(21.77 − x)CaO–xSrO by melt quench, with x ranging from 0 to 21.77 in mol%), and observed that the increase in the SrO content reduced the hydroxyapatite layer formed, with the absence of this layer being observed for glasses with levels greater than 10%. Also, the decrease in apatite formation was related to the slightly higher pH values and the formation of a secondary crystalline phase layer of apatite containing Sr, which has greater solubility in SBF when compared to typical Ca-apatite.

Similar results were observed by Sriranganathan et al. [89] who synthesized glasses (SiO_2_–P_2_O_5_–Na_2_O–K_2_O–CaO–SrO–MgO–NC) with 17.8 and 35.6 mol% Sr^2+^ using a high-temperature melt quench route and studied the dissolution of this powder and its ability to form apatite after immersion in Tris buffer (pH 7.4) and simulated body fluid (SBF). The results indicated that the replacement of Ca by Sr in bioactive glasses with high phosphate content can delay the formation of the apatite phase. The apatite formation, in this glass, occurred through a precursor phase of octacalcium phosphate (OCP) due to the low amount of Ca, which only subsequently transformed into apatite.

Sr-doped bioactive glasses with contents greater than 5 mol% Sr^2+^ produced via sol–gel also exhibited reduced apatite layer formation. In this sense, Wu et al. [60] reported that the doping of 5 mol% Sr^2+^ in amorphous 64S bioactive glasses (64SiO_2_–5P_2_O_5_–31CaO mol.%) produced via sol–gel promoted an inhibitory effect on the formation of the apatite-like compound, due to the formation of a secondary crystalline phase layer of apatite containing Sr^2+^, with extremely high solubility in SBF. Moghanian et al. [100] produced amorphous bioactive glasses doped with Sr^2+^ (60SiO_2_–(36 − x)CaO–4P_2_O_5_–xSrO, x = 0, 5 and 10 mol.%) via sol–gel and also observed that replacing Ca with Sr reduced the capacity for apatite formation. Hu et al. [124] also investigated the effect of the substitution of Ca by Sr on BG (SiO_2_–P_2_O_5_–CaO–SrO, Sr = 6 and 15 mol.%) produced via sol–gel, and verified a decrease in the amounts of Ca^2+^ ions released from the glass, which, added with PO_4_^3−^ from the SBF solution, are responsible for inducing apatite formation. Therefore, this decrease promotes a delay in the ability to form apatite in the presence of the Sr ion. Goudarzi et al. [125] also produced amorphous BGs (60SiO_2_–(36 − x)CaO–4P_2_O_5_–xSrO, x = 2, 4, 6 and 8 in mol.%) derived from sol–gel and showed that the hydroxyapatite formation rate in the sample containing 2% Sr^2+^ was higher than that of the other samples.

**Figure 3:**
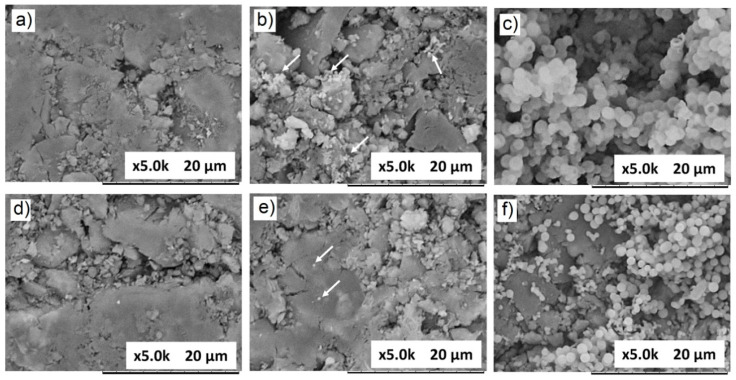
Images of BG 58S samples with 0% Sr^2+^ before (**a**) and after immersion in SBF for 7 (**b**) and 14 days (**c**); BG samples with 5% Sr^2+^ before (**d**) and after immersion in SBF for 7 (**e**) and 14 days (**f**). Reprint and adapted from reference [126], copyright (2022), with permission from Ceramics International.

More recently, Oliveira et al. [126] produced BG 58S (60SiO_2_–36CaO–4P_2_O_5_, mol%) with 0 and 5% Sr^2+^ via sol–gel and after immersion in SBF observed the formation of crystallized hydroxyapatite in both samples. However, the introduction of Sr ions into BG promoted a gradual reduction in apatite crystalline phases. Figure 3 shows the morphology of samples of BG 58S with 0% Sr^2+^ (Figure 3a–c) and with 5% Sr^2+^ (Figure 3d–f) before and after immersion in SBF for 7 and 14 days. Spherical particles (apatites) on the surface of the sample after 7 days of immersion in SBF were observed. It is also worth to highlight that bioactive glasses prepared without Sr exhibit a surface completely covered by hemispherical apatite particles after 14 days of immersion in SBF, while BG prepared with Sr exhibits a surface that is less covered by precipitates. On the other hand, Fiorilli et al. [102] produced amorphous BG of SiO_2_ and CaO with Sr incorporation (2 and 4 mol%) by aerosol-assisted spray drying and sol–gel methods and did not observe a significant influence on the kinetics of apatite formation caused by the amount of ion, when compared to pure BG samples.

Studies indicate that the presence of Sr^2+^ can promote a delay in the kinetics of apatite nucleation in BGs, according to the ion concentration and the production technique used, generally when used in an amount higher than 5%mol, but also showed that all the materials studied presented bioactive potential in in vitro analysis [102,105,117,126].

### 3.2. Osteogenic Differentiation

Osteoblasts are derived from undifferentiated mesenchymal stem cells (MSCs) and are responsible for the deposition of bone matrix in tissue. The production of osteoblasts from undifferentiated cells occurs after the osteoprogenitor stimulation that is dependent on transcription factors that control cell proliferation and maturation [127]. Bone formation carried out by osteoblasts is strongly linked to the process of bone resorption, which consists of the formation of a resorption pit that is filled by osteoblasts [128]. This process promotes an increase in Ca concentration at the site that induces the cell replication and the expression of osteogenic genes in osteoblasts mediated by the calcium-sensing receptor (CaSR) that are important for cell differentiation and survival [129].

Sr^2+^ can promote osteogenic differentiation or osteogenesis by joining CaRS, and activating proteins and genes involved in this process, which directly influences the acceleration of bone repair and regeneration [129]. According to the literature [14], the Sr^2+^ present in the composition of BGs is released gradually upon the dissolution of the glass, inhibiting the differentiation of osteoclasts and increasing osteoblastic activity, in addition to inducing the formation of hydroxyapatite without causing inflammation. Therefore, the addition of Sr^2+^ to the BG network has the potential of improving the proliferation and differentiation of pre-osteoblastic cells into osteoblasts.

Studies report that the amount of Sr^2+^ influences the osteogenic response [130,131]. Verberckmoes et al. [130] suggested that at very low concentrations, between 0.5 and 1 μg/mL, Sr^2+^ compromises the in vitro cell differentiation of osteoblasts. For concentrations between 2 and 5 μg/mL, a normal mineralization was observed, while for the highest doses of Sr^2+^ (20 and 100 μg/mL), a reduced mineralization was observed. All these observations were compared to the control group (0 μg/mL Sr^2+^). According to Aimaiti et al. [131], the addition of small amounts of Sr^2+^ (25–500 µM) promotes the osteogenic differentiation of human adipose tissue-derived stem cells (hASCs), while higher doses (1000 to 3000 µM) promote the apoptosis of hASCs.

Other previous studies have shown an increase in the osteoblastic differentiation of fetal mouse calvary cells when using bioactive glass particles (SiO_2_–CaO–SrO) containing 5 wt% of Sr^2+^ produced via sol–gel [132]. Likewise, more recent research has studied the osteogenic response of Sr-doped bioactive glasses in mesenchymal cells obtained from the bone marrow of rats and has shown better results for concentrations around 25 mol% of Sr^2+^.

In this study, Santocildes-Romero et al. [99] partially (50%) and completely (100%) substituted Ca with Sr in the 45S5 amorphous bioactive glass network using a melt quench route. Real-time PCR analyses detected the upregulation of genes associated with osteoblastic differentiation for all bioactive glass compositions. Also, genes such as Alpl and Bglap were stimulated in compositions with Sr^2+^. Glasses containing Sr^2+^ promoted osteogenesis in a differentiating bone cell culture model, with the composition containing 50% Sr exhibiting the highest levels of metabolic activity compared to controls. Li et al. [17] also studied the influence of Sr^2+^ concentration (between 5 and 25 mol%) on amorphous bioactive glasses. These researchers produced a series of borate-based glasses ((59 − x)B_2_O_3_ –3P_2_O_5_–13CaCO_3_–15Na_2_CO_3_–10TiO_2_–xSrCO_3_), and observed that glasses containing 20 and 25 mol% Sr^2+^ promoted osteoblastic cell proliferation (MC3T3-E1), while BGs containing lower levels inhibited growth. This trend was observed in bioactive glasses produced via sol–gel [133], where it was seen that increasing the Sr concentration up to 25 mol% promoted an improvement in the metabolic activity of osteoblasts in amorphous quaternary BGs (SiO_2_–CaO–SrO–P_2_O_5_), when Ca was partially and completely replaced by Sr^2+^ (0–100%). This study also observed a better ALP activity in Sr -doped glasses when compared to pure BG. However, increasing concentrations above 25% resulted in a decrease in ALP activity.

Studies carried out by Naruphontjirakul and collaborators [134,135,136] showed that the cell viability in the MTT of pre-osteoblastic cells MC3T3-E1 and hMSCs treated with Sr-doped BG nanoparticles, up to a concentration of 250 μg/mL, did not show significant changes when compared to the control. Initially, the researchers produced bioactive glass nanoparticles (Si_2_O–CaO–SrO) containing Sr using a modified sol–gel process and replaced Ca with 0 and 100 mol% Sr^2+^ [135]. Then, Naruphontjirakul et al. [136] evaluated the osteogenic response of a murine pre-osteoblastic cell line, MC3T3-E1, in the bioactive glass with the same composition and synthesis method as the previous study, introducing only 6 and 14 mol% of Sr^2+^. The immunohistochemical (IHC) staining of Col1a1, osteocalcin (OSC) and osteopontin (OSP) proteins in MC3T3-E1 cells demonstrated their expression after three weeks of culture. Since the markers of late osteogenic differentiation, OSC and OSP, are more clearly expressed by cells cultured in BG with 14 mol% SrO, this indicates that the presence of Sr accelerates mineralization without osteogenic supplements, stimulating osteogenic differentiation of the pre-osteoblastic cell line. It also shows that the Sr-doped BG did not change MC3T3-E1 cells’ viability up to a concentration of 250 μg/mL. Later, Naruphontjirakul et al. [134] showed that doping BG with 4.4 and 9.4 mol% of Sr^2+^ stimulates the differentiation of bone marrow-derived human stem cells (hMSCs) through an osteogenic pathway and accelerates mineralization, up to a concentration of 250 μg/mL. Furthermore, Sr-BGNP dissolution products did not adversely affect hMSC viability and no significant differences in viability were measured between each composition. However, the relative cell viabilities of particle concentrations above 500 µg/mL were all significantly reduced after seven days of culture.

In a more recent study, Naruphontjirakul et al. [137] produced BG monodispersed nanoparticles with 4.4 and 9.4 mol % Sr^2+^ and evaluated, using the WST-1 assay, the cell viability of macrophages isolated from murine bone marrow when exposed to samples for 24 h. An increase in cell viability was observed for all concentrations, when compared to cells cultured in basal medium, suggesting that macrophages became more metabolically active during their exposure to particles. Furthermore, it was also reported that when incubating RAW264.7 cells, the cells were polarized toward the pro-regenerative M2 population instead of the pro-inflammatory M1 population.

On the other hand, studies [95,100,102,103] using sol–gel BG glasses produced with levels below 5 mol% showed good differentiation results in MC3T3-E1 cells. In this sense, Moghanian et al. [100] produced 58S bioactive glasses (60SiO_2_–(36% − x)CaO–4P_2_O_5_–XSrO) doped with 5 and 10 mol% Sr^2+^ using sol–gel. The Live/Dead and DAPI/Actin color tests showed that the variation in Sr content promoted the proliferation of viable cells. And, they indicated that the composition with 5% mol of Sr, the lowest content used in the study, was indicated as ideal, as it was capable of improving the metabolic behavior of osteoblasts, increasing the cell proliferation and differentiation of MC3T3-E1 cells, and enhancing the alkaline phosphatase activity when compared to 0 and 10 mol% compositions.

**Figure 4:**
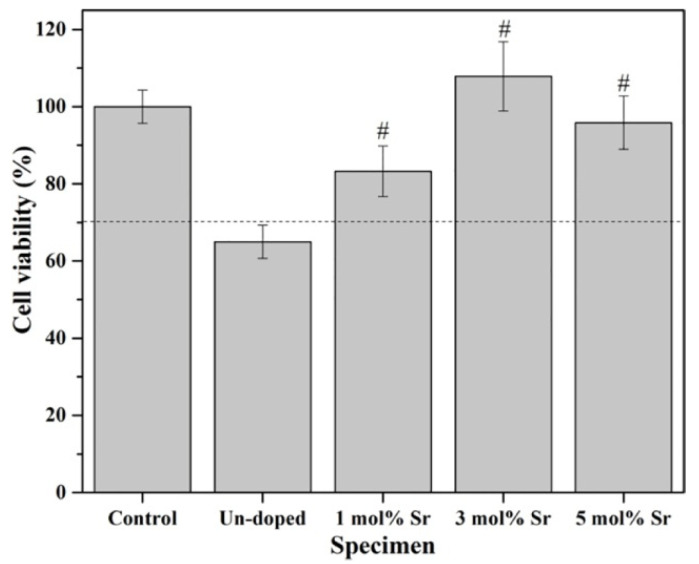
Cell viability of spray-dried un-doped, 1 mol%, 3 mol, and 5 mol% Sr-doped BG microspheres derived from MC3T3-E1 osteoblast cells after incubation for 3 d (#: *p*-value < 0.05). Reprinted from reference [95] copyright (2022), with permission from Journal of Non-Crystalline Solids.

Fiorilli et al. [102] showed that mesoporous bioactive glasses (SiO_2_–CaO) containing 2 mol% of Sr^2+^, produced by aerosol-assisted spray drying and sol–gel, are capable of stimulating the expression of pro-osteogenic genes, such as COLL1A1, SPARC and OPG, confirming the potential of the ion as a therapeutic element for stimulating bone remodeling. In addition, Swe et al. [103] produced, via sol–gel, a BG (SiO_2_–P_2_O_5_–Na_2_O–CaO–Al_2_O_3_–SrO) doped with 3 mol% Sr^2+^ and reported an improvement in the proliferation of MC3T3-E1 osteoblast-like cells, ALP activity and bone nodule formation when compared to pure BG. Also, using MC3T3-E1 osteoblastic cells, Ningsih et al. [95] produced microspheres of BG 58S (60SiO_2_–35CaO–5P_2_O_5_, mol%) doped with 1, 3 and 5 mol% Sr^2+^ using spray-drying and also evaluated cell viability. Their results showed that the cell viability of the undoped BG microsphere was lower (64.9%) than the standard ISO level of 70%, indicating toxicity. For BG doped with 1, 3 and 5 mol%, in turn, cell viability values of 83.3%, 107.9% and 95.9%, respectively, were verified. This indicates that Sr^2+^ improved the osteoblastic cells’ MC3T3-E1 cell viability when compared to the non-doped BG specimen, as shown in Figure 4. These results are confirmed by statistical tests that showed *p* values lower than 0.05 (indicated by # in the graph), with a significant difference between the doped and undoped samples. However, based on the data presented above, it is possible to conclude that levels below 5% mol Sr^2+^ produced better results.

In this context, Kumar et al. [138] produced BG S53P4 (53SiO_2_–23Na_2_O–15CaO–4P_2_O_5_–5SrO, mol%) mesopores doped with 5% Sr^2+^ by the evaporation-induced self-assembly (EISA) method. Increased calcium deposition in MG-63 cells, which are of osteoblast-like lineage, was observed, indicating mineralization. The alizarin red staining protocol dye confirmed the enhanced mineralization in the presence of osteogenic induction media after 14 days and raised the hypothesis that culture media containing Sr^2+^ can stimulate the osteogenic differentiation of MG-63 without the addition of osteogenic supplements. Therefore, the Sr^2+^ addition selectively affects osteoblasts, improving early development toward the osteoblastic phenotype while interfering with osteoclastogenesis.

Other important components for bone remodeling are osteoclasts, which are multinucleated cells responsible for reabsorption of the existing bone matrix [139]. For the differentiation, production and maturation of resorptive cells to occur, macrophage colony-stimulating factors (M-CSF) are required, in addition to the presence of RANKL, which is produced by osteoblasts [140]. Osteoclast precursors and mature osteoclasts also express CaSR in their cell membranes, and Ca detection may play an important role in controlling the function of these cells.

Sr^2+^ has a dual function in this sense, acting both by increasing osteoblastic differentiation and proliferation, as well as by reducing osteoclast differentiation and bone activity. For some years now, the role of Sr^2+^ in decreasing osteoclastogenesis has been reported, thus dominating the number of mature osteoclasts, regardless of the concentration used [141,142]. However, although much is said about these functions of Sr^2+^ in osteoclastogenesis reactions, there are still few studies that report the influence of this differentiation process when Sr is introduced through doped BGs.

Among the few reports, Gentleman et al. [143] studied the replacement of the Ca ion with the Sr ion up to 100% in the 46.46SiO_2_–1.07P_2_O_5_–26.38Na_2_O–23.08(SrO:CaO) (mol%) vitreous lattice produced via the melt quench route. The authors verified the inhibition of osteoclastic differentiation in experiments with cells in culture media treated with ionic dissolution products with concentrations ranging from 5 to 23 ppm. They attributed this fact to the decrease in tartrate-resistant acid phosphatase (TRAP) activity and the prevention of calcium phosphate film reabsorption. Fredholm et al. [68] produced, also via melt quench and a quenching process, glasses 49.46SiO_2_–1.07P_2_O_5_–(23.08 − x)CaO–xSrO–26.38Na_2_O (x= 0–23.08) (mol%) doped with Sr^2+^ and observed greater osteoblast fixation and osteoblast proliferation, in addition to inhibiting the differentiation and reabsorption of osteoclasts in relation to undoped BG. In order to investigate osteoclastic differentiation in BG doped with 6 mol% Sr^2+^, Wei et al. [22] carried out a study using the TRAP staining test, and observed the presence of TRAP-positive cells in doped and undoped BG. However, quantitative studies indicated a significant reduction in osteoclasts in Sr-doped BG.

During osteoporosis, the activity of osteoclasts exceeds that of osteoblasts and clinical ways to inhibit this activity are needed, sometimes using osteoclast bone resorption inhibitors such as alendronate sodium (ALN). However, its use presents a series of disadvantages and to minimize this fact, Zhao et al. [144] used BG nanoparticles doped with Sr^2+^ and ALN (A-SrBG). The glass was obtained via the sol–gel method and ALN was adsorbed on the surface. The A-SrBG nanoparticles produced had the ability to promote the differentiation of osteoblasts, while inhibiting the differentiation of osteoclasts, preventing RANKL-induced activation of the NF-κB and mevalonate pathways. When implanted in vivo, the A-SrBG samples promoted the regeneration of new bone. These results indicated that the samples may be a promising candidate for the reconstruction of bone defects in osteoporosis. Wu et al. [145] developed a Ca/Si bioactive glass containing Sr^2+^ (0 g, 0.67 g or 1.34 g) using a simple high-temperature method and also obtained a material with the ability to prevent osteoporosis while inducing osteogenesis, confirming the double mechanism promoted by Sr^2+^.

Therefore, more comprehensive studies are necessary to determine Sr^2+^ ion levels that allow the adequate regulation of osteoclast activity, allowing balanced absorption because the exaggerated increase in osteoclastic activity can cause, in addition to bone lysis, the rapid degradation of the biomaterial and new bone tissue. On the other hand, the low activity of these cells can promote excessive tissue formation and necrosis [146].

### 3.3. In Vivo Behavior

Three-dimensional tissue engineering structures must exert not only physical support [147], but must also improve cell adhesion, proliferation, migration and also cell differentiation for the proper healing and restoration of bone tissue functions.

Additionally, the combination of Sr with other ions in a porous BG structure promotes increased levels of bone regeneration in rabbit thigh injuries [148,149,150].

Studies (CITAr) have indicated the positive influence of Sr on the regenerative capacity of BG at concentrations below 10 wt% in in vivo studies. Gorustovich et al. [151] studied, for the first time, the in vivo behavior of 45S5 BG particles, produced by melt quench doped with 6 wt% SrO as a CaO substitute in rat tibia bone marrow implants. However, the results did not show a significant bone response when compared to pure BG. Years later, Wei et al. [22] incorporated Sr^2+^ into mesoporous BG (MBG: CaO–P_2_O_5_–SiO_2_) and observed an in vivo efficacy in the repair and healing of bone defects in rat femurs induced via an ovariectomy. The rats received skin incisions and the ovaries were removed, and after 8 weeks it was found that MBG with Sr regenerated 50% more new bone tissue when compared to pure MBG, indicating bone formation efficiency.

Furthermore, Zhang et al. [152] also showed that Sr-MBGs promoted alveolar bone defect regeneration in an osteoporotic animal model following a bilateral ovariectomy. MBG containing Sr^2+^ showed a greater formation of new bone tissue (46.67%) when compared to pure MBG. Also, Zhao et al. [93] produced strontium-containing mesoporous bioactive glass scaffolds (57.2SiO_2_–7.5P_2_O_5_–35.3(SrO + CaO)) by 3D printing and reported good osteogenic capability and apatite-forming ability, being able to stimulate MC3T3-E1 cell proliferation and differentiation and new blood vessel formation in critical-sized rat calvaria defects within 8 weeks.

In line with previous studies, more recently, Mosaddad et al. [71] performed histopathological and histomorphometric tests at 4, 8 and 12 weeks after the implantation of Sr-doped BG 45S5 scaffolds (with 0.05 g of Sr) in the skull and femur of rabbits. Histopathological test results evidenced high levels of bone formation, cell infiltration and proliferation when compared to the pure BG and control groups. Also, these results were corroborated by the histomorphometric test, which showed increases in bone regeneration levels in the Sr-BG group at weeks 8 and 12. Recently, Zhang et al. [104] also produced 45S5 bioactive glass doped with Sr^2+^ (5–20 mol.%) and applied it to ovariectomy bone marrow-derived mesenchymal stem cells (OVX-BMSCs). The results of a femoral condyle defects model in OVX rats indicated that Sr10/45S5 granules enhanced bone regeneration in osteoporotic bone defects through an early improvement in autophagy and late activation of the Akt/mTOR signaling pathway.

Barbeck et al. [9] prepared ICIE16 glass (48SiO_2_–6.6Na_2_O–32.9CaO–2.5P_2_O_5_–10K_2_O) doped with 5 wt% Sr^2+^ using the melt quench method, and performed an in vivo experiment subcutaneously in Sprague-Dawley rats. Their results indicated that the ICIE16-Sr bioactive glass induced a greater number of M1 macrophages after 30 days and a few M2 macrophages comparable to those found in the control groups, which did not significantly influence the foreign body response or the vascularization of the implantation bed in vivo.

**Figure 5:**
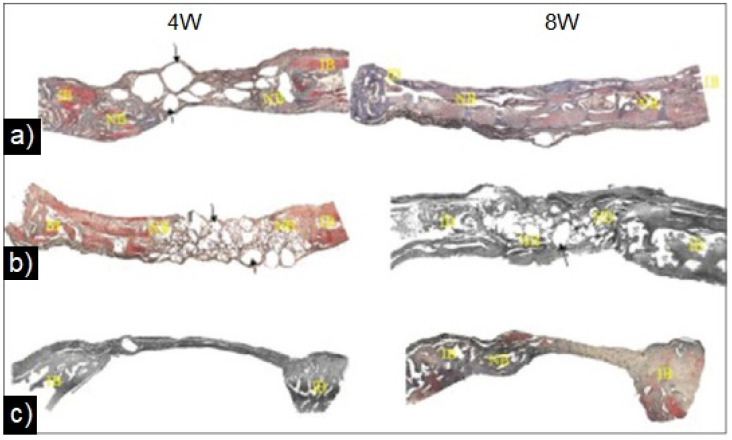
Comparison of newly formed bone (NB) in experimental groups. (**a**) Strontium-doped bioactive glass, (**b**) standard 45S5 bioactive glass and (**c**) unfilled defect (negative control). Reprinted and adapted from reference [153], copyright (2022), with permission from Dental Research Journal.

In the same year, Esfahanizadeh et al. [153] produced, via sol–gel, BG 45S5 doped with 5 mol% Sr^2+^ and investigated the influence of this filling on the amount of bone regeneration in critical defects of rabbit calvaria. It was observed that in the fourth and eighth week, Sr-doped bioactive glass demonstrated more new bone formation with rates of 11.1% and 28.32% of regenerated area/mm^2^, respectively. Also, at the fourth week, 55.38% of residual material was found in the defects filled with Sr-doped BG, which may be related to the initial phase of osteogenesis with a low percentage of absorbed material. At the eighth week, in turn, the lowest value of residual material among the samples was observed (12.9%). The histological examination performed (Figure 5) also showed a greater formation of new bone tissue in Sr-BG samples compared to pure BG samples. It is also possible to observe that the remainder of the Sr^2+^ granules surrounded by connective tissue are at the edge of the defect.

### 3.4. Antibacterial Properties

For appropriate bone regeneration, a strong chemical bond between the material and living tissue and the prevention of bacterial infection at the implant site are both important in order to avoid serious problems such as generalized infections, implant failure and even the need for a new surgery. Therefore, it is extremely important to evaluate the antibacterial properties of materials used in implants [14,154].

Despite the antibacterial activity of Sr^2+^ still not being widespread, it is already possible to observe that this ion can also exert an inhibitory effect on several bacterial strains. Sr can be introduced into the BG structure and be gradually released during glass dissolution, promoting the controlled delivery of this therapeutic ion [14].

Li et al. [17] studied the effects of Sr^2+^ on the antibacterial activity of borate-based bioactive glass produced by melt quench. They used six different formulations of the glass (59 – x)B_2_O_3_–3P_2_O_5_–13CaCO_3_–15Na_2_CO_3_–10TiO_2_–xSrCO_3_ (x= 5–25, mol.%), increasing the concentrations of SrCO_3_. It was observed that all glasses with concentrations of Sr^2+^ lower than 25 mol% were bacteriostatic against *Staphylococcus aureus* (*S. aureus*) in the short term of 1–7 days. This fact can be observed since the increase in Sr^2+^ doping slowed down the dissolution rate of BG due to the resistance of the ionic bond formed, blocking the release of other ions.

Another study involving BG incorporated with Sr^2+^ was developed by Manoochehri et al. [19], seeking new therapeutic approaches that refer to bone regeneration. In this study, glass based on SiO_2_/PO_4_/CaO and SrO was produced via the sol–gel method, and scaffolds were subsequently prepared by adding glass in amounts of 10%, 20% and 30% to an alginate and chitosan solution. The antibacterial activity of the scaffolds is shown more specifically against S. aureus and E. coli. Furthermore, the materials obtained maintained mechanical stability to guarantee the integrity of the replaced bone.

Also, the antibacterial effect of Sr-substituted 58S glass against methicillin-resistant *S. aureus* bacteria was evidenced in the work of Moghanian et al. [100]. The 58S (SiO_2_–CaO–P_2_O_5_–SrO) was produced using the sol–gel method with the replacement of Ca by Sr in contents of 0–10 mol%. In this study, the Sr^2+^ incorporation implied higher pH values that varied between 7.75 and 7.8 for Bg5 and Bg10 samples, respectively (against a pH of 7.6 exhibited for the sample without Sr^2+^), resulting in the bactericidal efficiency of the bioactive glass against methicillin-resistant *S. aureus* (MRSA). Based on other results, the low concentration of Sr^2+^ in the samples and the pH value induced better cell proliferation, although lower amounts of Sr^2+^ increased the solubility of Bg compared to samples without Sr^2+^.

Ranga et al. [16] studied the effect of introducing Sr^2+^ into the bioactive glass matrix (55SiO_2_–40CaO–(5 − x)SrO − xAg_2_O) (with x = 0, 2 and 4) produced using sol–gel. An antimicrobial study revealed that the prepared bioactive glass did not show an inhibition zone on *E. coli* or *S. aureus* with the presence of Sr^2+^. An antimicrobial effect was only observed in Ag-doped BG. Later, Baheiraei et al. [96] produced Sr-doped glasses (SiO_2_–CaO–SrO–P_2_O_5_) via sol–gel by replacing Ca with Sr^2+^ (at the percentage of 5 wt%) and reported that the ion improved the antibacterial activity against the same bacteria. The in vitro results showed complete inhibition of *E. coli* growth and partial inhibition of *S. aureus* growth, with a three-fold reduction in the growth index, using the counting method and colony-forming unit determined on Mueller–Hinton (MH) agar plates. It is reported that the antibacterial activity of Sr-doped BG may be related to the higher concentration of Ca, P and Sr ions released in SBF solutions and higher pH values when compared to pure BG samples. Araujo et al. [105] also reported the antimicrobial effect of 45S5 bioactive glass doped with 2 mol% Sr against *E. Coli* after an incubation time of 24 h at 37 °C. This behavior was attributed to an increase in the degradation rate of the samples, which also promotes an increase in pH due to the release of alkaline ions, implying an unsuitable surrounding environment for bacterial growth.

Therefore, according to reports [155,156], the antibacterial mechanism for BG may be related to the dissolution of ions, depending on their concentration, and a consequent increase in pH, creating an environment that allows a quick inhibition of bacterial growth.

## 4. Conclusions

This paper provides an overview of the influence of Sr^2+^ on the biological properties of BGs. And, despite the number of publications on the topic, the importance of specific research regarding Sr^2+^ concentrations to be inserted into BG networks is notable. This makes it possible to better evaluate the biological responses and release mechanisms of this ion since this material exhibits a good biological response for bone tissue engineering, due to its biocompatibility, bioactivity and acceptable mechanical properties. Furthermore, these materials promote enzymatic activity, vascularization, osteoblast adhesion, and the differentiation and proliferation of mesenchymal cells into osteoblasts.

It was also observed that the BG manufacturing method can alter its biological properties. For example, BGs doped with Sr^2+^ produced using melt quench enabled the retention of the amorphous structure after sintering, while sol–gel-derived glasses showed that an increase in ion content promoted an increase in the crystallization of the material. However, the introduction of Sr^2+^ into the network of BGs produced by both techniques resulted in excellent biological responses, and the antibacterial potential of these systems was also reported. This mechanism, still largely underexplored, can advance the development of new materials that promote bone regeneration and prevent infections, significantly accelerating the healing of bone lesions.

This ion is also known to be beneficial for the treatment of bone diseases such as osteoporosis due to its ability to change bioactivity—a function that is associated with increased adhesion, proliferation and cell differentiation—and to promote osteoblast function and inhibit osteoclast activity.

## Figures and Tables

**Table 1:** Summary of the reported biological functions of Sr-doped bioactive glasses.

Composition of Bioactive Glass (mol%)	Concentration (mol%)	Precursor ion Sr	Process	Sr Function in Bioactive Glass	Reference
45SiO_2_–24.5Na_2_O–24.5CaO–6P_2_O_5_	1	SrO	Hydrothermal	Improves the osteogenic ability and stimulates new blood vessel formation in critical-sized rat calvarial defects.	[93]
46SiO_2_–24.5Na_2_O–26.9(CaO + SrO)–2.6P_2_O_5_	50 and 100	SrO	Melt quench	Stimulates genes associated with osteoblastic differentiation (including *Alpl* e *Bglap)* and promotes osteogenesis in a differentiating model of bone cells culture.	[99]
(59 − x)B_2_O_3_–3P_2_O_5_–13CaCO_3_–15Na_2_CO_3_–10TiO_2_–xSrCO_3_	5–25	SrCO_3_	Sol–gel	Promotes osteoblastic cells (MC3T3-E1) proliferation (in concentrations of 20 and 25 mol%) and exhibits antibacterial effects against *S. aureus* in the short term (1–7 days).	[17]
60SiO_2_–36CaO–4P_2_O_5_	5 and 10	Sr(NO_3_)_2_	Sol–gel	Increases MC3T3-E1 differentiation, proliferation and ALP activity. Promotes the formation of hydroxycarbonate apatite on the BG surfaces and exhibits antibacterial effect against *S. aureus*.	[100]
18SiO_2–_36B_2_O_3–_22CaO–6Na_2_O–8K_2_O–8MgO–2P_2_O_5_	3, 6 and 9	SrO	Melt quench	Suppresses the fast release of boron and minimizes cytotoxicity. Also improves MG-63 cell proliferation.	[101]
SiO_2_–CaO	2 and 4	SrCl_2_	Aerosol-Assisted Spray Drying and Sol–gel	Stimulates the expression of pro-osteogenic genes, like COLL1A1, SPARC and OPG. Promotes apatite layer formation after 1 day of SBF immersion and does not significantly affect the surface ion-exchange kinetics.	[102]
55SiO_2_–40CaO–(5 − x)SrO–xAg_2_O	1, 3 and 5	_	Sol–gel	Positive effect on the materials’ bioactivity, promoting hydroxyapatite layer formation after 7 days of immersion in SBF.	[16]
53.85SiO_2_–1.72P_2_O_5_–21.77Na_2_O–(22.65 − x)CaO–1Al_2_O_3_–xSrO	3	Sr(NO_3_)_2_	Sol–gel	Formation of high density of needlelike shape HAp crystals 7 days after immersion in SBF. Increases MC3T3-E1 osteoblast-like cell proliferation, ALP activity and bone nodule formation.	[103]
45SiO_2_–6P_2_O_5_–24.5Na_2_O–(24.5 − x)CaO–xSrO	5, 10 and 20	SrO	Melt quench	Promotes the regeneration of osteoporotic bone defects via early improvement in autophagy and late activation of the Akt/mTOR signaling pathway.	[104]
45.2SiO_2_–2.5P_2_O_5_–23.9Na_2_O–26.4CaO–2Al_2_O_3_–2SrO	2	Sr(NO_3_)_2_	Melt quench	Inhibitory effects on *E. coli* and cell viability greater than 90% by using the NCTC clone 929.	[105]
60SiO_2_–35CaO–5P_2_O_5_	1, 3 and 5	Sr(NO_3_)_2_	Spray drying	Positive effect on the bioactivity and significant increase in osteoblast cell viability.	[95]
46.1SiO_2_–26.9CaO–2.6P_2_O_5–_24.35Na_2_O	1 and 2	Sr(NO_3_)_2_	Melt quench	Antibacterial activity against E. coli and a positive effect on the viability of Saos-2 cells (ATCC ® HTB-85™ human osteosarcoma cell line).	[98]
74SiO_2_*–*(26 − x) CaO–xSrO	1, 3 and 5	Sr(NO_3_)_2_	Sol–gel	Affects the microstructure of nanoparticles, resulting in a decrease in specific surface area on the pore size of nanoparticles and reduced polymerization of the glass network.	[106]

**Table 2:** Patents published with Sr-doped BGs in the last 10 years according to Google Patents—search topic “bioactive glass”, refined with “strontium”.

Title	Patent Request	Process	Description	BG (%mol)	Request Year	Reference
Preparation of strontium-containing bioglass powder and preparation method of strontium-containing porous bioglass bracket	CN 103848574 A	High-temperature melting	Repair, treatment and promotion of bone growth	40CaO–28SiO_2_–20Na_2_O–2K_2_O–2.5MgO–3P_2_O_5_–4B_2_O_3_–0.5SrO	2014	[107]
Bioactive glass with strontium added	P5469598	Sol–gel	BG for bone repair or reconstruction	45–75 SiO_2_–15–30 CaO– 2–8 SrO–0–10 P_2_O_5_ Other elements: 0–1	2014	[108]
A kind of 3D-printing bracket of mesoporous bioglass containing strontium of biological absorbable and preparation method thereof	CN 106267374 B	3D printing	Scaffolds using treatment for bone defect	57.2SiO_2_–7.5P_2_O_5_–35.3(SrO + CaO)	2016	[109]
Nanobioactive glass cement comprising strontium-doped bioactive glass nanoparticle and preparation method thereof	10-2439780	High-temperature melting	Apatite deposition and tissue regeneration	85SiO_2_–10CaO–5SrO	2019	[110]
Oral composition added with bioactive glass and strontium chloride and application thereof	CN 109431834 B	Oral composition	BG for oral hygiene with anti-allergy effect and tooth-defect-repair effect.	The bioactive glass and of the strontium chloride are both 0.5 to 15 wt%	2021	[111]

## Data Availability

Not applicable.
